# An Action Plan to Face the Challenge of Dementia: INTERNATIONAL STATEMENT ON DEMENTIA from IAP for Health

**DOI:** 10.14283/jpad.2018.27

**Published:** 2018-06-20

**Authors:** H. Chertkow, Research Executive Committee of the Canadian Consortium on Neurodegeneration in Aging, International IAP committee on Dementia

**Affiliations:** 1 3755 chemin de la Côte-Ste-Catherine, H3T 1E2, Montréal, Québec, Canada

**Keywords:** Risk reduction strategies, risk factors, life course, public awareness, national plans

## Abstract

An international committee set up through the IAP for Health met to develop an action plan for dementia. Comprehensive international and national initiatives should move forward with calls for action that include increased public awareness regarding brain health and dementia, support for a broad range of dementia research objectives, and investment in national health care systems to ensure timely competent person-centred care for individuals with dementia. The elements of such action plans should include: 1) Development of national plans including assessment of relevant lifecourse risk and protective factors; 2) Increased investments in national research programs on dementia with approximately 1% of the national annual cost of the disease invested; 3) Allocating funds to support a broad range of biomedical, clinical, and health service and systems research; 4) Institution of risk reduction strategies; 5) Building the required trained workforce (health care workers, teachers, and others) to deal with the dementia crisis; 6) Ensuring that it is possible to live well with dementia; and 7) Ensuring that all have access to prevention programs, care, and supportive living environments.

## Introduction

This statement has been prepared by an international committee set up through the InterAcademy Partnership for Health (IAP-H), which is a component of the The InterAcademy Partnership (IAP). The InterAcademy Partnership (IAP) was launched in 2016, and currently has a membership of 135 academies of science, medicine and engineering from around the world. These include both national academies/institutions as well as regional/global groupings of scientists. This statement is modified from a position paper that was initially commissioned by the Canadian Academy of Health Sciences, and written, approved and submitted by the Research Executive Committee (REC) of the Canadian Consortium on Neurodegeneration in Aging (CCNA). Members of the Research Executive Committee of the CCNA and the International IAP committee on Dementia also appear at the end of the statement.

The proportion of the world's population that is 65 years of age or greater has grown over the last number of decades, and this trend will continue. Advancing age is the greatest known risk factor for dementia (1, 2). If there is no change in age-standardized prevalence, societal aging is predicted to nearly triple the number of individuals living with dementia worldwide by 2050 (3, 4). It is estimated by that year the number of individuals with dementia will rise from 47.5 million people to an estimated 135.5 million with most of this increase occurring among people living in low- and middle income countries (2). Aside from the personal cost of dementia, these rising numbers will be associated with an economic burden. The 2015 global estimated cost of dementia was US $818 billion and is expected to be a trillion dollars by 2018 (5).

The World Health Organization (WHO) now recognizes dementia as a public health priority (6, 7). To respond to this challenge, a global series of actions initiated during the UK G8-Presidency in 2013 were undertaken by bodies such as the Organisation for Economic Co-operation and Development (OECD; (8)), Alzheimer's Disease International (ADI) and by the World Dementia Council (WDC; (9)).

## Dementia Overview

Dementia is an acquired, persisting and typically progressive decline in cognitive abilities, affecting learning and memory, language, and/or reasoning that is severe enough to interfere with independence in everyday activities. It becomes more common with increasing age during adulthood. Besides cognitive impairment, dementia is often associated with debilitating neuropsychiatric symptoms, such as agitation, psychosis, sleep disturbance, depression, anxiety and apathy (10). Dementia can arise from numerous conditions acting alone or in combination (11, 12). For many it is due to a neurodegenerative process, an umbrella term for a number of debilitating conditions that result in the progressive degeneration and/or death of neurons (5). Alzheimer disease is the most common neurodegenerative cause of dementia and is currently incurable. A mixture of brain diseases often underlies dementia, with many people showing changes consistent with both Alzheimer and cerebrovascular disease (13, 14). Dementia is usually a slowly progressive illness where the diagnosis is made after the process has been present for years (15).

Risk factors and conditions (such as smoking, or diabetes) commonly associated with vascular conditions (stroke, heart disease) are also known to be associated with dementia (16, 17). Frailty itself is a considerable risk factor for dementia (18). Parkinson's disease is closely associated with the development of dementia (19). The majority of older individuals with dementia have mixed pathology in their brain (11, 12, 20, 21).

While young onset (under 60 years) dementia is seen infrequently in many countries, this may not be the case in countries with high HIV prevalence. The HIV epidemic is concentrated in younger people of low-income countries, particularly in Sub-Saharan Africa, where young people may subsequently bear a disproportionately greater burden of dementia (22).

Women are at both greater risk of developing dementia and then living longer with the condition after its onset (23). Women also provide most of the informal (unpaid) care for people living with dementia.

While there are currently no cures for the neurodegenerative conditions that lead to dementia, emerging research suggests that some life-style factors (e.g., engaging in physical activity, managing blood pressure, selected forms of cognitive training) may have the potential to delay, if not prevent, its onset (24, 25, 26, 27). Population studies have suggested additional associated risk and protective factors, which require research to evaluate their potential as primary prevention intervention targets (28, 29). The progress to date in developing effective pharmacological treatment options has been disappointing (30, 31, 32), underscoring the need to understand better what contributes to the dementia syndrome in different generational cohorts as well as in different populations (33).

A key area for research and support is the development and dissemination of improvements for the care provided to people living with dementia including compassionate and appropriate end-of-life care (34, 35, 36, 37). Greater acceptance and inclusion of people living with dementia within communities is increasingly seen as an important factor in improving their quality of life and minimizing disability (7, 38). The needs of patients and their families change along the course of dementing illnesses and it is necessary to gear support and therapy for the different stages of the disease.

## A Call to Action

Because of these issues, developing a comprehensive strategy internationally to address the challenges of dementia will require wide consultation followed by the long term implementation of a comprehensive, integrated and responsive series of actions. Most initiatives will be nationally based, but additional international collaborations to address dementia will also be advantageous. The nationally based initiatives will generally share similar high-level goals and principles to address this global health problem. We call for countries within regions that have resources, to establish a network that can support other countries similar to them in their approach to dementia. The goals and principles of a call to action would include addressing the following broad areas: (a) Increasing public awareness - educating the general population about dementia, how to maintain brain health, and on the importance of addressing this health challenge, accepting people with dementia as they are, and accommodating to their remaining abilities; (b) supporting fundamental research to find and implement effective approaches (both pharmacological and non-pharmacological) to delay, prevent, slow down, treat, ameliorate, and eventually cure the common causes of dementia; (c) investing in national health care systems — this would entail both training a sufficient number and mix of providers as well as building the necessary infrastructure to ensure timely, competent person-centered care is available to those living with dementia and their caregivers through all stages of the illness.

Our Call to Action is one which aims at developing an evidence-based and a public health orientated approach. Ultimately, this should include a clear assessment for each population of the potential for primary prevention (upstream prevention), secondary prevention (early detection followed by effective treatment, considered to be likely more effective at that stage than later) and tertiary prevention (mitigation of dementia and its ramifications through various therapies and end-of-life care for those with dementia).

## Elements of an Action Plan to Face the Challenge of Dementia

An action plan to face the challenge of dementia in its global context must include a concerted and coordinated series of actions from policies, to research, to care, to social inclusion. Here are seven key elements of such an action plan.

### National dementia plans must be established

National plans to combat dementia have been initiated in 29 countries/states since 2005. There is a global plan on dementia being developed by the WHO (6, 7) and the first regional plan on dementia in the Americas, published by the Pan American Health Organization (PAHO) in October 2015 (See the website of ADI https://www.alz.co.uk/dementia-plans/ for a list of national plans currently underway as well as countries currently lacking national plans). Canada is the only G7 country without a national dementia plan (39).

Each country should develop a national plan coherent with its health care goals which could coordinate activities, harmonize where appropriate with international efforts, promote the sharing of successful local initiatives, address identified gaps, ensure efficient use of resources, and mobilize further investment in all aspects of dementia including care and research. A national plan would acknowledge dementia as a public health priority and heighten awareness of this daunting health challenge.

As a first step, towards such plans, we propose that a national dementia status report should be carried out in as many countries with resources as possible. Such a status report for each region would be wide-ranging, including burden of all dementia types, comorbid disorders, risk factors, therapeutic approaches and care systems.

More research is necessary to establish the strength and interaction of lifecourse risk and protective factors relevant to dementia. Nevertheless, assessment of the “exposome”, potential risk and protective factors for each population, would be an important part of this report (40, 41). These should establish, for key lifestages, the balance in those populations of positive and negative features for brain health (42, 43). This would encompass a broad range of environmental factors such as maternal health, early life health, infections, education, vaccination, as well as adverse exposures such as poor housing, smoking, poor diet, and exposure to noxious substances.

A 5-year follow-up report should be planned to document the impact of national policies (public awareness, risk factors, care systems, etc.) and the creation of a national dementia strategy.

### Increase investment in national research programs on dementia

The investment in medical research varies widely across countries. In 2016 the American investment in dementia research was US $936 million, which translates to US$2.93 per capita (23). In contrast, the Canadian investment in research on dementia was smaller (less than a quarter per capita of what is invested in the USA) (44). Overall, developed countries do not adequately invest in dementia research when compared to the funding of research on other conditions such as cancer and heart disease even though the cost of caring for persons with dementia is estimated to be greater than that for dealing with either of the other two conditions (45, 46). It has been stated that a goal of 1% of the national annual cost of dementia should be steered into dementia research programs (Dementia in Canada: A National Strategy for Dementia-friendly Communities, Report from Canadian Senate, 2016 (6)). This additional investment in each country will have to be thoughtfully allocated and managed. Broad coordination within each country should be organized for best use of research funds. Governance and prioritization of dispersal of these funds must also involve individuals living with dementia and their caregivers, the research community, and practitioners.

### This investment must span all aspects of dementia research

Allocated research funds should support a broad range of activity from biomedical investigation to inquiries dealing with clinical aspects, health systems and services research. There must be fundamental research to unveil the mechanisms involved in the onset of neurodegenerative diseases and hopefully pave the way to a specific and effective pharmacological treatment. In addition, research to gain better insight into understanding the social cultural and environmental factors that affect the health of populations is essential. Investments should target national research capacity, supporting knowledge transfer, addressing the needs of unique populations (for example, indigenous people and those living in rural and remote communities (47, 48, 49)), investigating sex and gender differences in dementing conditions, and embracing ethical and social dimensions (50, 51).

There is now considerable potential for earlier diagnosis of various forms of dementia using clinical, imaging, and biomarker support (52). The advantages and potential of early diagnosis is a critical focus of research in Western countries (53, 54, 55, 56, 57). Attention must now be paid to delineating the optimal approaches to early diagnosis and establishing the risks and benefits of translating this knowledge into health care policy.

The neuropsychiatric (behavioral and psychological) symptoms of dementia need more attention given their strong impact on quality of life, caregiver burden and rate of institutionalization (10, 58, 59, 60). Future research into the prevalence, etiology and therapy (including randomized controlled trials) of neuropsychiatric symptoms of dementia is needed.

There must be research investment into understanding what combinations of modifiable lifestyle factors across the lifecourse increase and decrease the risk, of developing dementia with aging (26, 61). This is not a one-size-fits-all syndrome across the globe. The combinations of relevant risk factors may vary in different cultures and communities. The most effective preventative and public health strategy for dementia will only emerge when the fullest understanding of these factors is achieved.

Specific attention should be devoted to the support of social research aimed at identifying the actual needs of subjects with dementia and their caregivers (62, 63, 64). The general purpose of such investigations would be the planning of multifaceted interventions encompassing environmental, psychological, medical and social support.

### Risk reduction strategies should be instituted

While there is still a considerable amount to learn about the full interplay of risks, governments must support national risk reduction and empowerment strategies for the public and support the efforts of health professionals to promote healthy brain aging. Current evidence can be used to empower the public and health professionals to act in ways that will reduce the risks of all dementia types developing, postponing the appearance of their clinical manifestations, and optimizing everyday functioning in meaningful social activities and roles. The focus of such risk reduction would include treatment and prevention of vascular risk factors — hypertension, obesity, diabetes, smoking, and high-calorie diets, and treatment of HIV to prevent HIV-related dementia. It would also include risk reduction to address sleep problems, illiteracy, head trauma, malnutrition, and physical inactivity in addition to other region-specific risk factors (5).

Risk reduction at the individual level must be supplemented by evidence-based structural and legislative alterations that support these reductions. Smoking legislation, strategies for excessive alcohol risk reduction, reduction of dietary salt, legislation to reduce head injuries are only a few of the risk reduction strategies that can be undertaken by governments to affect the occurrence of dementia in the population. Such governmental interventions will lead to less inequality because they benefit the disadvantaged as well. The WHO Global Noncommunicable Diseases Action Plan 2013–2020 focuses on many of these elements (6).

### The required workforce must be planned and trained

Workforce requirements to deal with the increasing number of persons with dementia must be determined and steps taken to ensure the required workforce is both trained and supported in their activities. A well trained and supported workforce of the right mix and number to deal with the needs of this emerging population is required. In each country, a national workforce plan will have to be created and implemented with the active involvement of local and regional authorities.

The full breadth of necessary trainees will only emerge after appropriate evidence-based strategies for risk reduction emerge. The workforce trained will initially be focused on the elderly, and the health care sector, but addressing modifiable risks (for example, limited education, early childhood nutrition) implies an investment in teachers, nutritionists, and a host of other professionals in the future.

### We must ensure that it is possible to live well with dementia

When a diagnosis of dementia is made, an individual should not be constrained to abandon her/his social role and participation. Creating the conditions within a country where one can live well with dementia includes ensuring that the public is aware of dementia in all its complexity, that there are accommodations in the environment (including work) to compensate for changing abilities, that there is adequate protection against abuses of all kinds against individuals living with dementia, and legal that rights are not automatically withdrawn from people living with dementia. Cooperation between academies and local administration should be encouraged so that all the needs of persons living with dementia and of their caregivers can be assessed and met.

### Access to prevention and care should be made available to all

To the extent possible, access to preventive programs, systems of care, and supportive living environments should be made available to all citizens with, or at risk of, dementia (49, 65, 66, 67)

## The Future of the Dementia Challenge

Dementia will be part of the global landscapes for many decades, reaching levels that are at least twice the current 2016 values. Indeed, even if research could provide the means of eradicating brain diseases causing dementia tomorrow, numerous individuals would already be on the trajectory to dementia. Brain diseases causing dementia are now known to start many decades before any clinical signs. For these reasons, a total solution will not be available for some time to come. This is why the member Academies of IAP for Health are focusing attention on the necessity of engaging in an action plan for dementia which is balanced and designed to address all aspects of the challenge, especially the wellness of those living with dementia and their caregivers.

*Conflict of interest:* Dr. Chertkow reports grants from the Canadian Institutes of Health Research (Foundation grant) and the Weston Foundation (Canada). He also reports clinical trial conduct-related fees from TauRx, Hoffmann-Laroche, and Merck Inc. In addition, he reports indirect support from the Alzheimer Society of Canada (funding partner of the Canadian Consortium on Neurodegeneration in Aging). No other disclosures relevant to the manuscript.
